# Anal human papillomavirus infection and its relationship with abnormal anal cytology among MSM with or without HIV infection in Japan

**DOI:** 10.1038/s41598-021-98720-3

**Published:** 2021-09-28

**Authors:** Daisuke Shiojiri, Daisuke Mizushima, Misao Takano, Koji Watanabe, Naokatsu Ando, Haruka Uemura, Yasuaki Yanagawa, Takahiro Aoki, Junko Tanuma, Kunihisa Tsukada, Katsuji Teruya, Yoshimi Kikuchi, Hiroyuki Gatanaga, Shinichi Oka

**Affiliations:** 1 AIDS Clinical Center, National Center for Global Health and Medicine, 1-21-1, Toyama, Shinjuku-ku, Tokyo, 192-8655 Japan; 2 Joint Research Center for Human Retrovirus Infection, Kumamoto University, Kumamoto, Japan

**Keywords:** Human papilloma virus, HIV infections

## Abstract

Anal high-risk human papillomavirus (hr-HPV) infection is widely considered a cause of anal cancer. However, epidemiological data are quite limited in Japan. This study investigated anal HPV infections and cytological abnormalities among MSM with or without HIV infection. Anal swabs were obtained, and cytological results were examined. Hybrid capture-based methodology was used for hr-HPV genotyping. The exclusion criterion was a history of vaccination against HPV. 644 subjects participated, and the overall prevalence of hr-HPV was 59.7% (95% CI 54.7–62.3), HIV-infected had higher prevalence than HIV-uninfected (68.9% vs 40.6%) *p* < .001. Among hr-HPV-infected participants, 82.8% (312/377) were infected with at least one of 9 valent vaccine-covered hr-HPV genotypes. From regression analysis, detection of abnormal cytology correlated positively with HIV infection (OR 2.17 [95% CI 1.51–3.13]), number of hr-HPV genotypes infected (OR 1.83 [1.59–2.10]), history of STI (OR 1.58 [1.14–2.22]) and No. of lifetime sexual partners (OR 1.56 [1.10–2.21]), albeit multivariate analysis identified the number of detected hr-HPV genotypes (adjusted OR 1.78 [1.54–2.06]) as the independent risk factor for abnormal cytology. High rates of anal hr-HPV infection, especially 9-valent HPV vaccine-preventable hr-HPV were detected among our MSM participants in Japan. HPV vaccination should also be encouraged for MSM in Japan.

## Introduction

Anal chronic infection by high-risk types of human papillomavirus (hr-HPV) and HPV-related malignancies are a growing concern of public health among men who have sex with men (MSM). Indeed, the prevalence of hr-HPV is extremely high not only among MSM with anal cancer but also among asymptomatic MSM^1,2^, and hr-HPV positivity and the risk of anal cancer are even greater in HIV-infected individuals^3–5^. Anal cancer screening using anal cytology has recently been performed in a few developed countries^6^, and in such countries, HPV vaccination is recommended for all male and female adolescents (from 9 to 26 years). Moreover, the Advisory Committee on Immunization Practices (ACIPs) in 2019 recommended shared clinical decision-making regarding HPV vaccination for some adults aged 27 through 45 years who are not adequately vaccinated^7,8^, even though preventive effects of vaccination against anal cancer or new acquisition of hr-HPV in sexually active adult populations have not been clearly demonstrated.

In Japan, the HPV vaccine for male individuals was approved only very recently, whereas genotyping of anal hr-HPV and anal-canal cancer screening are not currently approved. Furthermore, epidemiological data for MSM, including prevalent hr-HPV genotypes and related histopathological abnormalities, are quite limited, despite preliminary data suggesting high prevalence^9^.

In the present study, we assessed the epidemiology of anal colonization by hr-HPV and HPV-related abnormal cytology among HIV-infected and uninfected MSM and sought to discuss future strategies to prevent HPV-related anal cancer in Japan.

## Results

### Characteristics of the study participants

We enrolled a total of 644 MSM, including 437 HIV-infected and 207 HIV-uninfected eligible consenting participants. The demographic characteristics according to HIV-infected and uninfected subjects are presented in Table 1. The median age among HIV-infected and -uninfected participants was 46 (Interquartile range (IQR) 40–53) and 36 (IQR 29–43) years, respectively (*p* < 0.001).

**Table 1:** Demographic characteristics of HIV-infected and -uninfected Japanese MSM.

	HIV infected N = 437 Median (IQR) or N (%)	HIV uninfected N = 207 Median (IQR) or N (%)	*p* value
Age, years	46 (40–53)	36 (29–43)	< 0.001
No. of lifetime sexual partners	50 (20–100)	6 (3–15)	< 0.001
No. of sex partners in the past 6 months	2 (0–5)	3 (0–10)	< 0.001
% use of condom	70 (50–100)	70 (30–95)	0.256
**Past history of STIs**	317 (72.5%)	72 (34.8%)	< 0.001
Syphilis	222 (50.8%)	54 (26.1%)	< 0.001
Genital/anal warts	90 (20.6%)	2 (1.0%)	< 0.001
Genital/oral/anal gonorrhea	69 (15.8%)	7 (3.4%)	< 0.001
Genital/oral/anal chlamydia	63 (14.4%)	14 (6.8%)	0.006
Oral/genital/anal herpes	43 (9.8%)	2 (1.0%)	< 0.001
Latest CD4+ T-cell count × 10^9^/L	666 (512–821)	NA	NA
HIV-RNA < 20 copies/mL	427 (97.7%)	NA	NA
Any hr-HPV positivity	293 (68.9%)	84 (40.6%)	< 0.001

The denominator for hr-HPV positivity is 425.

*MSM* men who have sex with men, *STIs* sexually transmitted infections, *IQR* interquartile range, *hr-HPV* high-risk types of human papillomavirus, *UD* undetectable.

Compared to HIV-uninfected subjects, infected subjects had a significantly larger number of sexual partners in their lifetime but had fewer sexual partners in the past 6 months. There was no significant difference in the percentage use of condoms during anal sex in either group, but a history of STIs was significantly higher in HIV-infected than -uninfected subjects. All HIV-infected individuals were being treated with anti-retroviral therapy (ART), and plasma HIV RNA was undetectable (< 20 copies/mL) in 97.7% (427/437). For viremic patients, viral load was in the range of 21–119 copies/mL. CD4 counts were high, with a median of 666 × 10^9^/L (IQR 512–821).

### Prevalence of human papilloma virus

**Figure 1:**
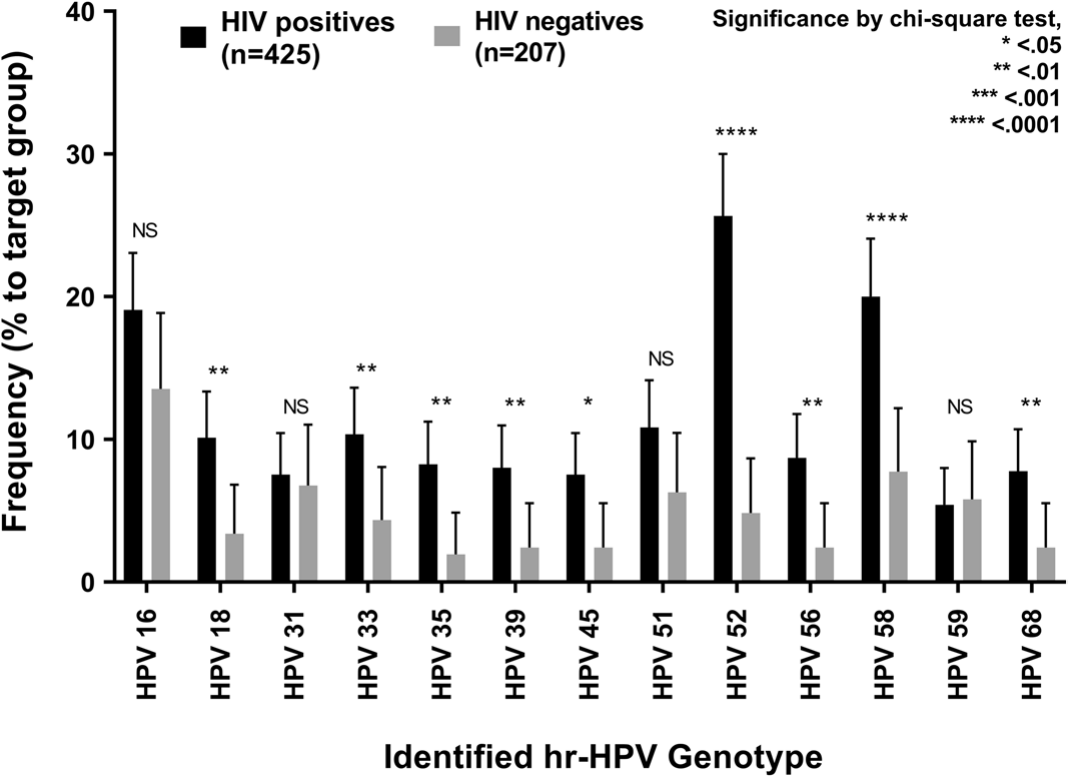
Prevalence of hr-HPV genotypes in HIV-infected and -uninfected MSM. The frequency of each hr-HPV genotype detected between HIV-infected and -uninfected subjects was compared. *HPV* human papillomavirus, *hr-HPV* high-risk types of human papillomavirus.

**Figure 2:**
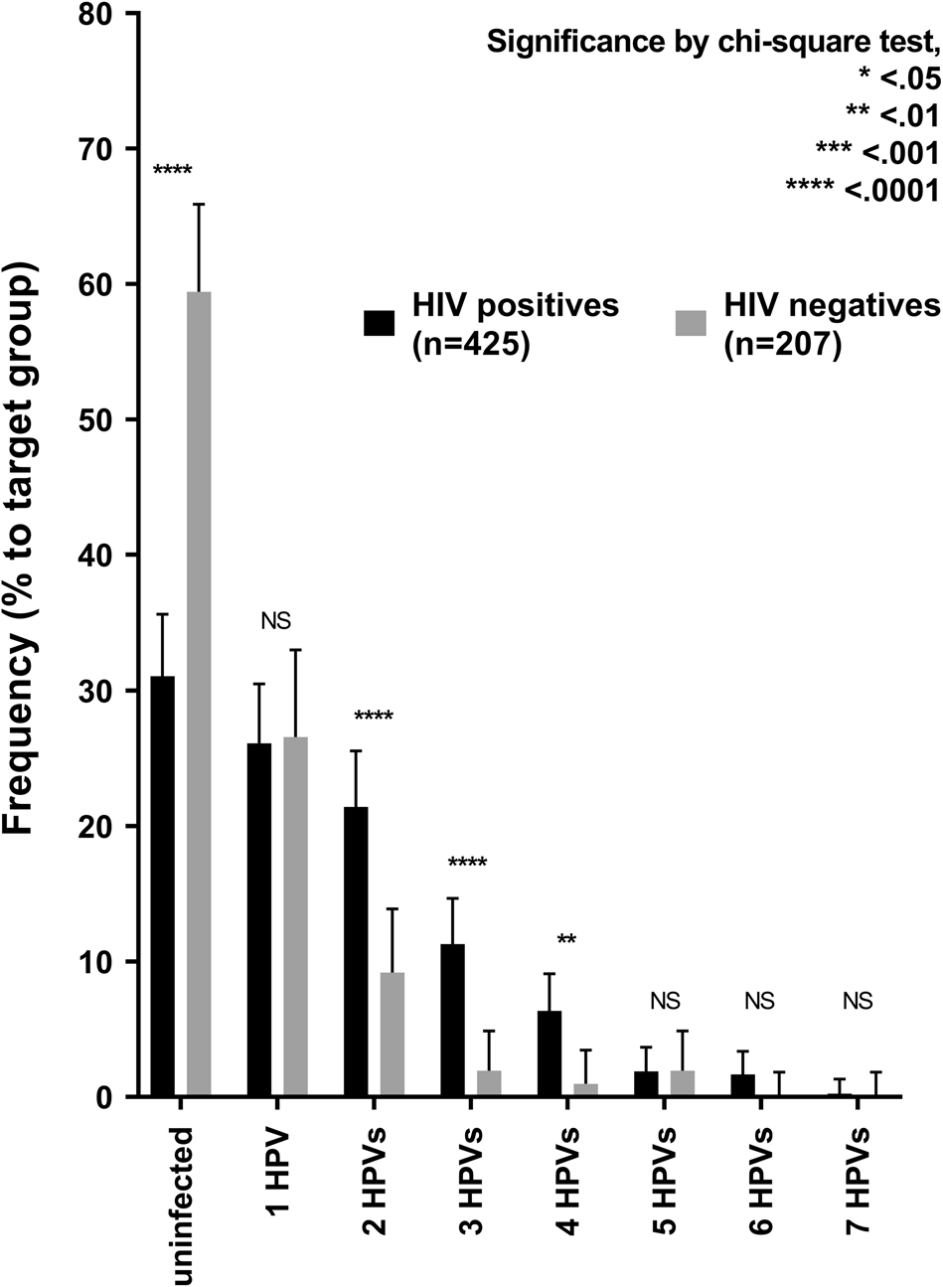
Number of hr-HPV genotypes in HIV-infected and -uninfected MSM. The frequency of hr-HPV genotypes identified between HIV-infected and -uninfected subjects was compared. *HPV* human papillomavirus, *hr-HPV* high-risk types of human papillomavirus.

Among the participants, sufficient anal samples for hr-HPV genotyping were available for 632/644 (98.1%) (425 HIV infected and 207 HIV uninfected). Figure 1 displays the distribution of infection with different hr-HPV genotypes in both HIV-infected and -uninfected patients. The overall rate of hr-HPV positivity was 59.7% [95% confidence interval (CI) 55.8–63.4], with HIV-infected MSM (68.9% [95% CI 64.4–73.2]) having a significantly higher rate than HIV-uninfected MSM (40.6% [95% CI 34.1–47.4]). The most significant difference between HIV-infected and -uninfected individuals was found for HPV52 and -58 (*p* < 0.001); HPV16, -52 and -58 were the most common HPV genotypes. The number of hr-HPV genotypes found in each participant stratified by HIV serostatus is illustrated in Fig. 2. Although there were more participants without hr-HPV infection among HIV-uninfected individuals than HIV-infected individuals (123/207 (59.4%) vs 132/425 (31.1%) (*p* < 0.001)), subjects with multiple infections were more prevalent among the latter than the former (182/425 (42.8%) vs 29/207 (14.0%) (*p* < 0.001).

Additionally, we observed that 312 of 377 h-HPV-infected subjects (82.8%), including 246 HIV-infected (84.0%) and 66 HIV-uninfected (78.6%) participants (*p* = 0.249), carried at least one of 9 valent vaccine-covered hr-HPV genotypes (genotypes 16, 18, 31, 33, 45, 52, and 58) (see Supplementary Table S1 online). When assessing according to age group in all subjects, most genotypes were equally prevalent, however, HPV52 and HPV56 prevalence rates tend to increase significantly with age (*p* = 0.0081 and *p* = 0.0058 respectively), (see Supplementary Figure S1 online).

### Cytology results

**Figure 3:**
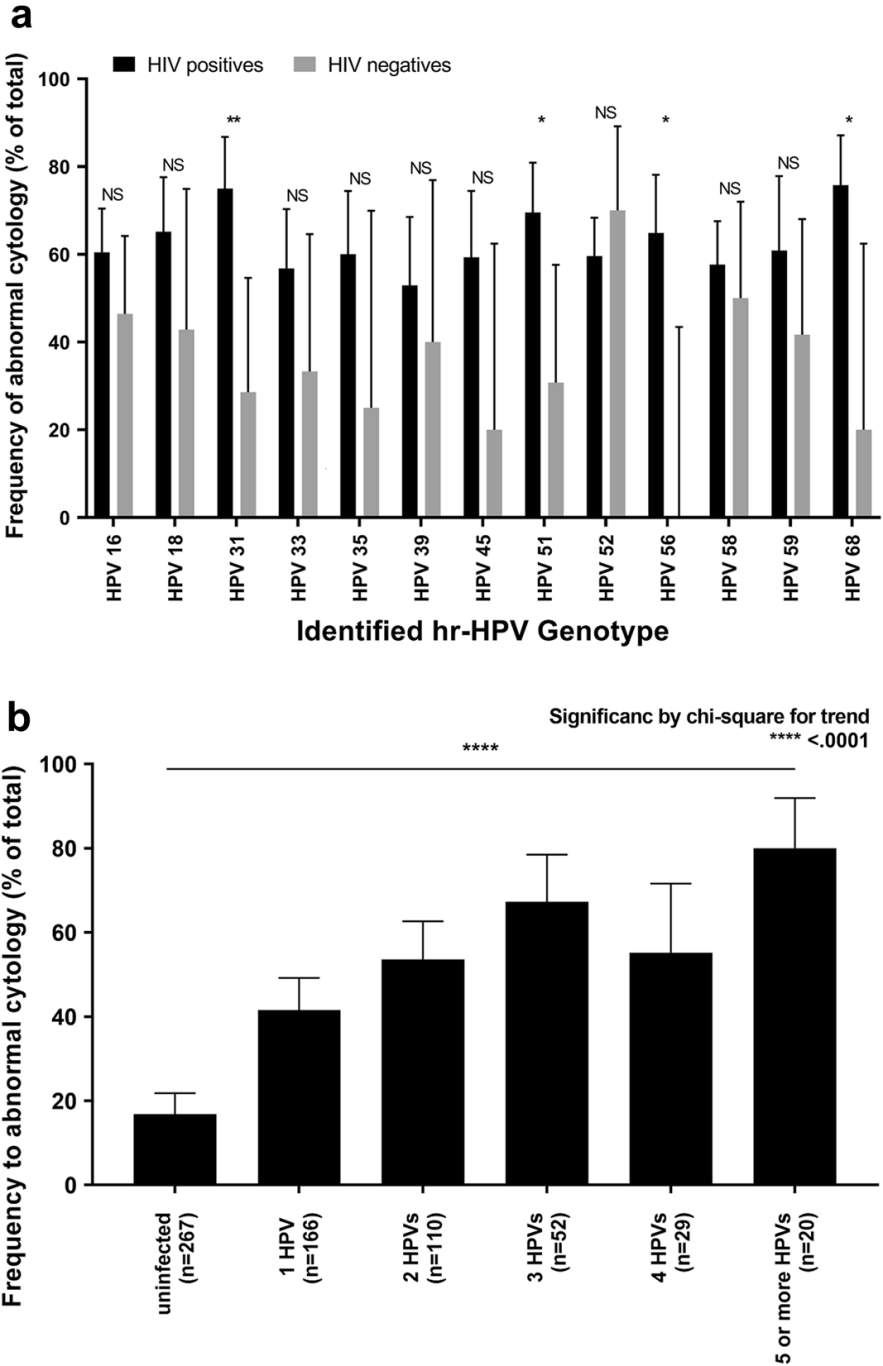
Frequency of abnormal anal cytology via anal swabs in HIV-infected and -uninfected patients. (**a**) The frequency of abnormal anal cytology results (ASC-US, LSIL, ASC-H, or HSIL) is presented in accordance with each hr-HPV genotype in both subjects (644). (**b**) The frequency of abnormal cytology results was calculated in accordance with the number of hr-HPV genotypes found in each subject (see Supplementary Table S2 for stratification by HIV serostatus). *HPV* human papillomavirus, *hr-HPV* high-risk types of human papillomavirus.

Cytology results were available for all participants, with sufficient anal samples for 644 (437 HIV infected and 207 HIV uninfected) subjects. Findings among participants with NILM (negative for intraepithelial lesion or malignancy), termed as normal cytology, or ASCUS + (atypical squamous cell of undetermined significance), termed as abnormal cytology, are shown in Table 2. Despite no age difference between participants with normal or abnormal anal cytology, HIV-infected MSM more often had abnormal anal cytology. Overall, we found no difference in the number of sexual partners in the past 6 months or in the percentage use of condoms during receptive anal sex in either group. Participants with abnormal anal cytology showed a prevalence of 25.8% and 12.9% for HPV16 and HPV18 infection, respectively, compared to 11.6% and 4.7% for those with normal anal cytology (*p* < 0.001). The prevalence of all hr-HPV types was 45.0% (182/404) for normal anal cytology, 79.2% (152/192) for ASC-US, 94.4% (17/18) for LSIL, 94.4% (17/18) for ASC-H, and 75.0% (9/12) for HSIL. Anal abnormal cytology results were observed in 37.3% (240/644) of all participants, with more cases among HIV-infected individuals (187/437; 42.8%) than HIV-uninfected individuals (53/207; 25.6%) (*p* < 0.001). Next, we assessed the impact of hr-HPV genotype on abnormal anal cytology between HIV-infected and -uninfected participants. Figure 3a depicts the frequency of anal abnormal cytology results based on each hr-HPV genotype. Compared to HIV-uninfected individuals, HIV-infected individuals had a significantly higher prevalence of anal abnormal cytology overall (86.6% versus 62.3%, *p* < 0.001), but with a significant difference only detected for HPV31, -51, -56 and -68. Correlation between the frequency of anal abnormal cytology and the number of hr-HPV genotypes detected in a subject is shown in Fig. 3b, and there was a significant chi-square trend for all subjects with abnormal cytology (*p* < 0.001) (Supplementary Table S2 shows stratification of the result by HIV serostatus).

**Table 2:** Findings among participants with NILM and ASCUS +.

	NILM, N = 404 Median (IQR) or N (%)	ASCUS + , N = 240 Median (IQR) or N (%)	*p* value
Age, years	43 (34–51)	45 (37–51)	0.065
Positive HIV status	250 (61.9%)	187 (77.9%)	< 0.001
No. of lifetime sexual partners	20 (4–80)	30 (10–100)	0.361
No. of sex partners in the past 6 months	2 (0–5)	2 (0–7)	0.367
% use of condoms during receptive anal sex	70 (40–100)	70 (40–97.5)	0.463
Past history of STIs	229 (56.7%)	162 (67.5%)	0.128
Number of subjects with detected high-risk HPV genotypes	182 (45.0%)	195 (81.3%)	< 0.001
Detection of HPV16	47 (11.6%)	62 (25.8%)	< 0.001
Detection of HPV18	19 (4.7%)	31 (12.9%)	< 0.001

*NILM* negative for intraepithelial lesion or malignancy, *ASC-US* atypical squamous cell of undetermined significance, *MSM* men who have sex with men, *STIs* sexually transmitted infections, *IQR* interquartile range, *hr-HPV* high-risk types of human papillomavirus.

### Risk factors for abnormal cytology

Finally, univariate and multivariate regression analyses were performed to identify factors affecting development of abnormal cytology. In univariate analysis, HIV infection (OR 2.17; 95% CI 1.51–3.13), number of detected hr-HPV genotypes (OR 1.83; 95% CI 1.59–2.10), past treatment history of STI (OR 1.58; 95% CI 1.14–2.22) and No. of lifetime sexual partners (OR 1.56; 95% CI 1.10–2.21) were significantly associated with abnormal cytology results, as indicated in Table 3. In multivariate analysis, HIV infection, history of STI and No. of lifetime sexual partners did not show independent risk factors for abnormal cytology, but the number of infected hr-HPV genotypes showed strong significance (aOR 1.78; 95% CI 1.54–2.06], *p* < 0.001).

**Table 3:** Multiple logistic regression analysis to determine factors associated with abnormal anal cytology.

All subjects (n = 632)	Univariate analysis		Multivariate analysis	
	OR [95% CI]	*p* value	aOR [95% CI]	*p* value
Age (+ 1 year)	1.01 [0.99–1.02]	0.166		
HIV infection	2.17 [1.51–3.13]	< 0.001	1.34 [0.88–2.04]	0.175
Number of detected hr-HPV genotypes	1.83 [1.59–2.10]	< 0.001	1.78 [1.54–2.06]	< 0.001
Past treatment history of STI	1.58 [1.14–2.22]	0.007	1.27 [0.86–1.86]	0.231
No. of lifetime sexual partners	1.56 [1.10–2.21]	0.013	1.00 [0.99–1.01	0.975
100% use of condom	0.86 [0.59–1.25]	0.432		

## Discussion

Anal HPV infection and its contribution to the risk of anal cancer is a growing concern, especially among MSM. However, epidemiological data remain limited for MSM in Japan, regardless of their HIV serostatus. Our study was performed at one of the largest healthcare centers for the HIV-infected population in Tokyo, and 90% of HIV seropositive individuals in Japan are MSM. The center also has the largest sexual health clinic for HIV-seronegative MSM in Japan as of now, where regular check-ups for HIV and STI testing, as well as HIV pre- and post-exposure prophylaxis, are carried out. Therefore, this study should generally represent a large proportion of MSM in eastern Japan. The results of the study showed that the prevalence of anal hr-HPV infection among all participants was 59.7% [95% CI 55.8–63.4] and that the most frequently detected hr-HPV genotypes were HPV-52 (18.8%), HPV-16 (17.2%), and HPV-58 (16.0%). Our previous study conducted 10 years ago in HIV-infected MSM identified a prevalence of hr-HPV infection as high as 75.9%, and the most frequent hr-HPV genotypes were HPV-58 (30.2%), HPV-16 (28.8%) and HPV-52 (22.2%)^9^. These 3 genotypes might be the predominant hr-HPV genotypes among Japanese MSM during the last decade. In this study, the prevalence of anal hr-HPV among HIV-infected subjects was 68.9% [95% CI 64.4–73.2], with a lower rate of 40.6% for HIV-uninfected subjects [95% CI 34.1–47.4]. The high prevalence of hr-HPV in HIV-seropositive MSM is similar to reports for other Asian countries, but the distribution of hr-HPV genotypes differs somewhat^4,10,11^. For example, the prevalence of hr-HPV infection is reportedly 85.2% in Taiwan, 82.7% in China and 85% in Thailand, and the most frequent hr-HPV genotypes are HPV-16, HPV-51 and HPV-52 in Taiwan and Thailand and HPV-16 and HPV-18 in China^4,10,11^.

In terms of anal cytology, the prevalence of hr-HPV was 45% for normal anal cytology, 79.2% for ASC-US, 94.4% for both LSIL and ASC-H, and 75.0% for HSIL. These findings are consistent with previous reports^10^. In addition, HIV-infected individuals were found to carry more hr-HPV genotypes than HIV-uninfected individuals and had an increased risk of abnormal anal cytology. Moreover, this study clearly documents for the first time in Japan that the prevalence of abnormal cytology correlates positively with the number of hr-HPV genotypes detected (Fig. 3b), as was discussed in a previous study^10^. This correlation was even stronger in HIV-infected than -uninfected, as shown in Supplementary Table S2. On the other hand, the prevalence of abnormal cytology was not greatly influenced by the difference in genotype (Fig. 3a). Furthermore, 82.8% (312/377) of subjects infected with hr-HPV (246 HIV infected and 66 HIV uninfected), harbored at least one of the 9 valent vaccine-covered hr-HPV genotypes, suggesting that HPV vaccination should be recommended, regardless of HIV serostatus and even to those who are already infected with some hr-HPV genotypes. Such measures would prevent acquisition of vaccine-preventable hr-HPV genotypes, as indicated previously^12^. Another associated risk factor for abnormal cytology was HIV infection, though among our participants, HIV infection was well controlled by antiretroviral therapy (ART). Nevertheless, it is not clear from the present cross-sectional study whether HIV infection preceding hr-HPV infection causes impaired clearance of hr-HPV infection or whether HIV-infected individuals re-acquire hr-HPV more readily.

There are some limitations to be considered in the present study. First, this was a single-site, clinic-based, cross-sectional study in Tokyo that does not represent the entire MSM population in Japan. Second, the study did not assess whether a case of hr-HPV infection was a transient infection, a persistent infection, or a reinfection. Third, this study only analyzed hr-HPV genotypes; therefore, not all hr-HPV genotypes were assessed. Finally, cytology results for HIV-uninfected subjects were reported only through a Pap smear and then translated to the Bethesda system using a previously reported relationship for the two systems^13^.

In conclusion, we found that hr-HPV, especially 9-valent HPV vaccine-preventable hr-HPV genotypes, is highly prevalent among MSM in Japan. Our study showed that an increased number of hr-HPV genotypes infected, is strongly associated with development of anal abnormal cytology; thus, HPV vaccination for Japanese MSM, those already infected or not, should be considered to prevent further acquisition of more hr-HPV genotypes, that would lead to HPV related anal malignancy. Further studies are needed to assess the health impact and cost-effectiveness of nine-valent HPV vaccination.

## Methods

### Ethics statement

Written informed consent for HPV and anal cytology testing was obtained from all participants. This study was approved by the ethics committee of the National Center for Global Health and Medicine (approval no. NCGM-G-002390). This study was implemented in accordance with the provisions of the Declaration of Helsinki.

### Study population

There is an existing group of HIV-infected MSM being treated at AIDS Clinical Center (ACC), National Center for Global Health and Medicine (NCGM), Tokyo, as well as a non-HIV-infected MSM group at the Sexual Health Clinic (SHC) outpatient in the same center. We conducted a cross-sectional study in these two groups between January 1, 2019, and March 31, 2019, for HIV-uninfected individuals and June 1 to August 31, 2019, for HIV-infected individuals. Eligibility criteria included male sex, age ≥ 16 years from SHC and age ≥ 20 years from ACC, self-reported history of having anal receptive sex with men, willingness to undergo anal swab sampling to test for hr-HPV infection, and anal cytology. Exclusion criteria was a history of HPV vaccination.

### Data collection

Each participant completed a standardized questionnaire. The information gathered included sociodemographic data (age), sexual behavior data (number of lifetime sexual partners, number of sexual partners in the last 6 months, percentage use of condoms (using measurement scales of 0 to 100, with intervals of 10), and history of sexually transmitted infection (including genital/oral/anal chlamydia, gonorrhea and herpes, syphilis, and genital/anal warts).

### Anal specimen collection for HPV genotyping and cytology

Samples were collected at the enrollment visit by well-trained health-care professionals using *digene*® specimen collection swabs, with a 5 cm insertion into the anal canal and a slow 360-degree rotating extraction. The swab was then shaken in BD SurePath liquid-based cytology fluid, and all samples were processed at the external laboratory LSI Medience Corporation Tokyo, Japan. HPV DNA was detected by hybrid capture-based methodology, as described previously^14^, which included only oncogenic or hr-HPV genotypes (16, 18, 31, 33, 35, 39, 45, 51, 52, 56, 58, 59, and 68)^15^. Anal cytology slides were interpreted by two cytopathologists blinded to the HIV or HPV infection status. Liquid-based cytology was prepared for routine Pap staining. The cytology results were reported first using Papanicolaou Classification (Class I–V)^16^ and also reported using Bethesda system classification categories, as follows: NILM, ASC-US, LSIL (low-grade squamous intraepithelial lesion), ASC-H (atypical squamous cells, cannot exclude high-grade lesion) and HSIL (high-grade squamous intraepithelial lesions)^16^. Slides with discordant interpretations were reviewed again under a multihead microscope to reach an agreement. Cytology results for HIV-uninfected subjects were reported only using Pap smears and then translated to the Bethesda system using the previously reported relationship of the two systems^13^; results for HIV-infected subjects were reported using both Pap smears and the Bethesda system.

### Statistical analysis

All statistical tests were evaluated at a 0.05 level of significance. Statistical analyses were performed with GraphPad Prism 7.0 and regression analysis with Statistical Package for Social Sciences (SPSS) software, version 23.0. We report categorical data as numbers (n) and percentages (%) and continuous variables as median and interquartile range (IQR). We compared differences in sociodemographics and sexual behaviors between HIV-infected and uninfected subjects and performed descriptive analysis. Mann–Whitney U tests were employed for continuous variables, and Chi-squared/Fisher's exact tests were used for categorical variables. The odds ratios (ORs) of significant factors associated with hr-HPV infection and the detection of anal abnormal cytological lesions were calculated using logistic regression analysis. Multivariate logistic regression models were then applied to the significant factors (*p* < 0.05) in univariate analysis.

## Supplementary Information

Supplementary Information 1.

Supplementary Information 2.
