# Supplementary material for: Anal human papillomavirus infection and its relationship with abnormal anal cytology among MSM with or without HIV infection in Japan
Source: Sci Rep. 2021 Sep 28;11:19257. doi: 10.1038/s41598-021-98720-3 (PMC8479121; doi:10.1038/s41598-021-98720-3)
Supplement: Supplementary file 1 — Supplementary Information 1. [file 41598_2021_98720_MOESM1_ESM.pptx]

## Slide 1
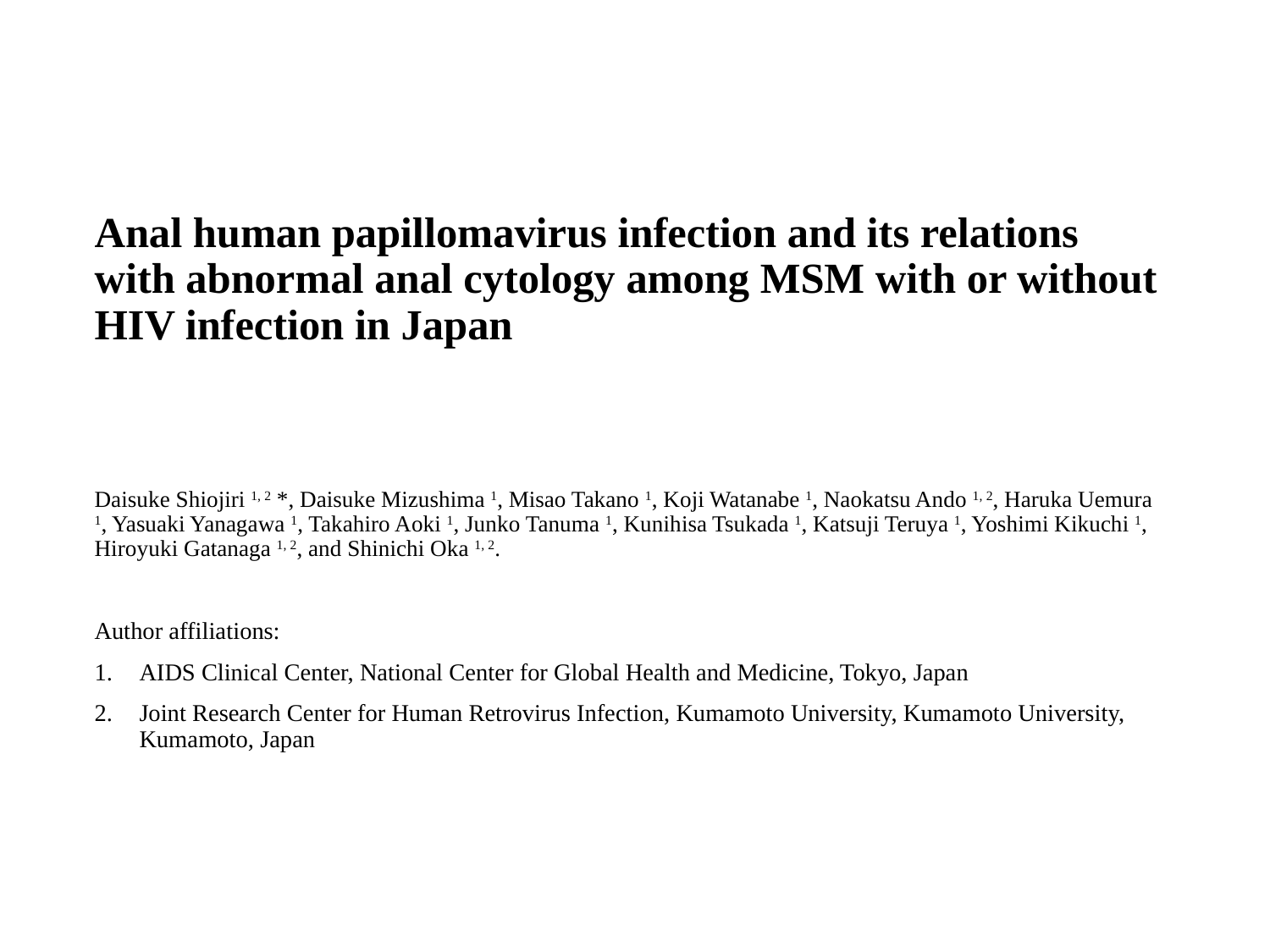

Anal human papillomavirus infection and its relations with abnormal anal cytology among MSM with or without HIV infection in Japan
Daisuke Shiojiri 1, 2 *, Daisuke Mizushima 1, Misao Takano 1, Koji Watanabe 1, Naokatsu Ando 1, 2, Haruka Uemura 1, Yasuaki Yanagawa 1, Takahiro Aoki 1, Junko Tanuma 1, Kunihisa Tsukada 1, Katsuji Teruya 1, Yoshimi Kikuchi 1, Hiroyuki Gatanaga 1, 2, and Shinichi Oka 1, 2.
Author affiliations:
AIDS Clinical Center, National Center for Global Health and Medicine, Tokyo, Japan
Joint Research Center for Human Retrovirus Infection, Kumamoto University, Kumamoto University, Kumamoto, Japan

## Slide 2
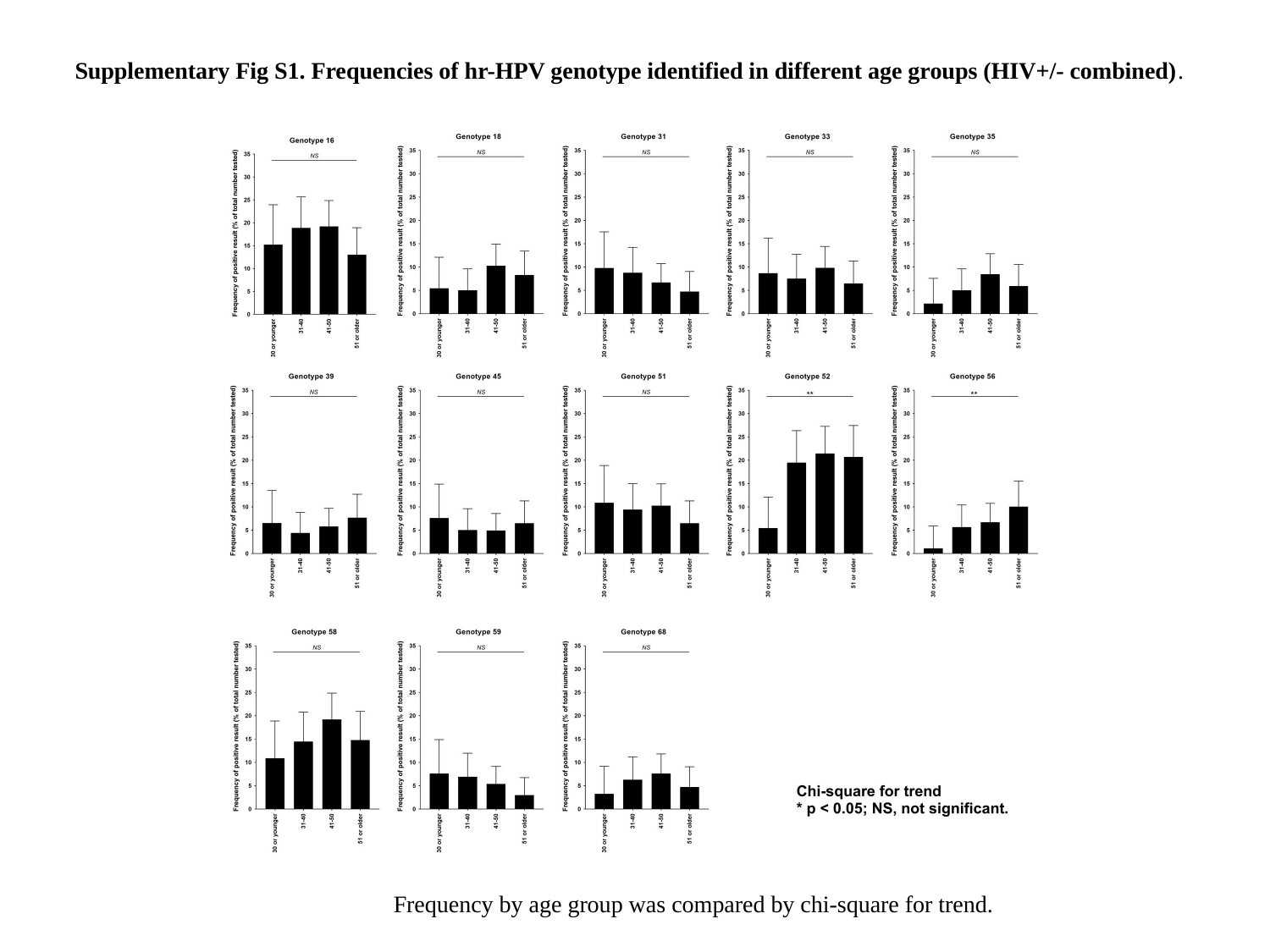

Supplementary Fig S1. Frequencies of hr-HPV genotype identified in different age groups (HIV+/- combined).
Frequency by age group was compared by chi-square for trend.
