# Supplementary material for: Anal human papillomavirus infection and its relationship with abnormal anal cytology among MSM with or without HIV infection in Japan
Source: Sci Rep. 2021 Sep 28;11:19257. doi: 10.1038/s41598-021-98720-3 (PMC8479121; doi:10.1038/s41598-021-98720-3)
Supplement: Supplementary file 2 — Supplementary Information 2. [file 41598_2021_98720_MOESM2_ESM.docx]

**Anal human papillomavirus infection and its relations with abnormal anal cytology among MSM with or without HIV infection in Japan**

Daisuke Shiojiri ^1, 2^ *, Daisuke Mizushima ^1^, Misao Takano ^1^, Koji Watanabe ^1^, Naokatsu Ando ^1, 2^, Haruka Uemura ^1^, Yasuaki Yanagawa ^1^, Takahiro Aoki ^1^, Junko Tanuma ^1^, Kunihisa Tsukada ^1^, Katsuji Teruya ^1^, Yoshimi Kikuchi ^1^, Hiroyuki Gatanaga ^1, 2^, and Shinichi Oka ^1, 2^.

**Author affiliations:**

^1^ AIDS Clinical Center, National Center for Global Health and Medicine, Tokyo, Japan

^2^ Joint Research Center for Human Retrovirus Infection, Kumamoto University, Kumamoto University, Kumamoto, Japan

**Supplementary Table S1. Vaccine preventable hr-HPV genotypes in HIV-infected and uninfected MSM in Japan**

| **Vaccine preventable hr-HPV genotypes** | **Overall N=632**  **N (%)** | | **HIV-infected N=425**  **N (%)** | **HIV-uninfected N=207**  **N (%)** | **P value** |
| --- | --- | --- | --- | --- | --- |
| Any hr-HPV genotype | 377 (59.7) | | 293 (68.9) | 84 (40.6) | <.001 |
| 2/4 valent hr-HPV genotypes | 148 (23.4) | | 119 (28.0) | 29 (14.0) | .219 |
| 9 valent hr-HPV genotypes | 312 (49.4) | | 246 (57.9) | 66 (31.9) | <.001 |
| HPV16 | 109 (17.2) | | 81 (19.1) | 28 (13.5) | 0.084 |
| HPV18 | 50 (7.9) | | 43 (10.1) | 7 (3.4) | 0.003 |
| HPV31 | 46 (7.3) | | 32 (7.5) | 14 (6.8) | 0.728 |
| HPV33 | 53 (8.4) | | 44 (10.4) | 9 (4.3) | 0.011 |
| HPV45 | 37 (5.9) | | 32(7.5) | 5 (2.4) | 0.01 |
| HPV52 | 119 (18.8) | | 109 (25.6) | 10 (4.8) | <.001 |
| HPV58 | 101 (16.0) | | 85 (20.0) | 16 (7.7) | <.001 |
| Other hr-HPV genotypes | |  |  |  |  |
| HPV35 | 39 (6.2) | | 35 (8.2) | 4 (1.9) | 0.002 |
| HPV39 | 39 (6.2) | | 34 (8.0) | 5 (2.4) | 0.006 |
| HPV51 | 59 (9.3) | | 46 (10.8) | 13 (6.3) | 0.065 |
| HPV56 | 42 (6.6) | | 37 (8.7) | 5 (2.4) | 0.003 |
| HPV59 | 35 (5.5) | | 23 (5.4) | 12 (5.8) | 0.842 |
| HPV68 | 38 (6.0) | | 33 (7.8) | 5 (2.4) | 0.008 |

**Abbreviations: MSM, men having sex with men; hr-HPV, high-risk types of human papillomavirus;**

**Supplementary Table S2. Frequency of abnormal cytology in accordance with the number of hr-HPV genotypes found in both HIV-infected and -uninfected MSM in Japan**

| Number of HPV | **Total abnormal cytology % (abn cytology /Normal+abn)** | **HIV-infected**  **% (abn cytology /Normal+abn)** | **HIV-uninfected**  **% (abn cytology /Normal+abn)** | **P value** |
| --- | --- | --- | --- | --- |
| Uninfected | 16.9 (45/267) | 17.4 (25/144) | 16.3 (20/123) | 0.811 |
| 1 HPV | 41.6 (69/166) | 42.3 (47/111) | 40.0 (22/55) | 0.773 |
| 2 HPVs | 53.6 (59/110) | 57.1 (52/91) | 36.8 (7/19) | 0.107 |
| 3 HPVs | 67.3 (35/52) | 70.8 (34/48) | 25.0 (1/4) | 0.060 |
| 4 HPVs | 55.2 (16/29) | 51.9 (14/27) | 100.0 (2/2) | 0.186 |
| 5 HPVs | 80.0 (16/20) | 93.8 (15/16) | 25.0 (1/4) | 0.002 |

**Abbreviations: MSM, men having sex with men; hr-HPV, high-risk types of human papillomavirus; abn cytology, abnormal cytology.**
